# RETRACTION: MATN1‐AS1 promotes glioma progression by functioning as ceRNA of miR‐200b/c/429 to regulate CHD1 expression

**DOI:** 10.1111/cpr.13767

**Published:** 2024-10-20

## Abstract

ZhuJ. , GuWT. , and YuC. , “MATN1‐AS1 Promotes Glioma Progression by Functioning as ceRNA of miR‐200b/c/429 to Regulate CHD1 Expression,” Cell Proliferation 53, no. 1 (2020): e12700. 10.1111/cpr.12700.31667976 PMC6985690

The above article, published online on 30 October 2019, in Wiley Online Library (wileyonlinelibrary.com), and has been retracted by agreement between the authors; the journal Deputy Editor, Yunfeng Lin; and John Wiley & Sons Ltd. The authors contacted the journal and reported that they had detected mistakes in the figures that compromised the validity of the article's results and requested a retraction. In addition, a report from a third party found duplications between Figures 2E and 2C with other articles by different authors, each of which describe different experimental conditions. Following further correspondence, the authors acknowledged these duplications and have stated that they cannot confirm the authenticity of the data. The retraction has been agreed to because the duplications of images across different articles fundamentally compromises the conclusions and results presented in the article.

J. Zhu , WT. Gu , and C. Yu , “MATN1‐AS1 Promotes Glioma Progression by Functioning as ceRNA of miR‐200b/c/429 to Regulate CHD1 Expression,” Cell Proliferation 53, no. 1 (2020): e12700. 10.1111/cpr.12700.31667976 PMC6985690

The above article, published online on 30 October 2019, in Wiley Online Library (wileyonlinelibrary.com), and has been retracted by agreement between the authors; the journal Deputy Editor, Yunfeng Lin; and John Wiley & Sons Ltd. The authors contacted the journal and reported that they had detected mistakes in the figures that compromised the validity of the article's results and requested a retraction. In addition, a report from a third party found duplications between Figures 2E and 2C with other articles by different authors, each of which describe different experimental conditions. Following further correspondence, the authors acknowledged these duplications and have stated that they cannot confirm the authenticity of the data. The retraction has been agreed to because the duplications of images across different articles fundamentally compromises the conclusions and results presented in the article.

